# Optimal l-arginine dosage for pressure injury healing in home care settings: a six-month retrospective analysis

**DOI:** 10.3389/fpubh.2025.1618928

**Published:** 2025-09-01

**Authors:** Nai-Ching Chen, Lihui Pu, Tai-Yang Huang, Chia-Shu Lin, Kuo-Chuan Hung, Yu-Yu Li

**Affiliations:** ^1^Department of Nursing, Chi Mei Medical Center, Tainan, Taiwan; ^2^Department of Internal Medicine, Section Nursing Science, Erasmus University Medical Centre, Rotterdam, Netherlands; ^3^School of Medicine, College of Medicine, National Sun Yat-sen University, Kaohsiung City, Taiwan; ^4^Department of Anesthesiology, Chi Mei Medical Center, Tainan, Taiwan; ^5^Department of Anesthesiology, Chi Mei Medical Center, Chiali, Tainan City, Taiwan; ^6^Department of Leisure and Sports Management, CTBC University of Technology, Tainan City, Taiwan

**Keywords:** pressure injuries, l-arginine supplementation, wound healing, home care, nutritional intervention, retrospective study

## Abstract

**Background:**

Pressure injuries present a significant challenge for patients receiving home care, particularly those with limited mobility. Although l-arginine supplementation has shown promise for wound healing, the optimal dosage remains unclear. This study compared the effectiveness of two daily l-arginine doses (7 g versus 14 g) in treating pressure injuries over a six-month period.

**Methods:**

We retrospectively reviewed the medical records of 120 adult patients with grade 3 or 4 pressure injuries who received home care between June and December 2023. Patient wounds were evaluated using the DESIGN-R assessment tool, which measures various aspects of wound healing. Statistical analysis was performed using SPSS 26.0, employing generalized estimating equations (GEE) to account for repeated measurements over time.

**Results:**

The study found that patients receiving the 7 g daily l-arginine dose showed significantly better wound healing after 1 month compared to those receiving 14 g (*p* < 0.001). While healing rates varied throughout the study period, the 7 g dose consistently demonstrated superior outcomes by the six-month mark (*p* < 0.05). Notably, the higher 14 g dose did not provide additional healing benefits at any point during the study.

**Conclusion:**

Our findings indicate that a daily 7 g l-arginine supplement effectively promotes both early- and late-stage healing of pressure injuries in home-care patients. This lower dose not only matches or exceeds the healing outcomes of the higher dose, but also presents a more cost-effective treatment option. Healthcare providers may consider implementing a 7 g dosage as a standard protocol for pressure injury treatment in home care settings.

## 1 Introduction

Pressure injuries, also known as pressure ulcers, are localized injuries to the skin and underlying soft tissue that typically occur over bony prominences because of prolonged pressure, shear forces, or both (1, 2). These wounds represent a significant healthcare challenge, with an estimated prevalence of 3.4–18.6% (3–5). This risk is particularly heightened in home care settings (6, 7), where limited resources and professional medical supervision can affect wound management efficacy. The development of pressure injuries strongly correlates with both immobility and nutritional status (8). Current evidence indicates that nutritional deficiencies significantly impede the healing process (9, 10), leading to a negative impact on patients’ quality of life and increased healthcare costs (11, 12). l-arginine has emerged as a promising nutritional intervention for pressure injuries owing to its multifaceted role in wound healing mechanisms (13), primarily as a precursor to nitric oxide, which enhances blood flow, oxygen delivery, and inflammation regulation in compromised tissues (14). Beyond vasodilation effects, it also significantly contributes to collagen synthesis and immune function enhancement, making it particularly valuable for patients with impaired healing processes, such as those with diabetes or chronic conditions (15, 16).

The therapeutic benefits of l-arginine in wound healing have prompted investigations into its optimal dosing regimen, a critical consideration for maximizing efficacy while minimizing potential side effects and costs. This dosage question is especially relevant in pressure injury management, where the treatment duration is typically extended and resources may be limited, particularly in home care settings. Establishing the most effective dose is essential not only for clinical outcomes but also for developing cost-effective treatment protocols that can be sustainably implemented across various healthcare environments. l-arginine supplementation at 4.5-9 g daily has demonstrated efficacy in enhancing wound closure rates across both malnourished and adequately nourished patients (17). Though a recent meta-analysis (18) suggested that higher doses exceeding 15 g might be beneficial, these conclusions were drawn from just two small studies with brief follow-up periods (i.e., 2–3 weeks) and limited patient samples. Given these constraints and the particular importance of cost-effectiveness in home care settings, more robust research with extended follow-up is needed to establish optimal l-arginine dosing protocols for pressure injury treatment.

To address this knowledge gap, this retrospective study aimed to evaluate and compare the effectiveness of two different l-arginine dosage regimens (7 g vs. 14 g daily) in promoting pressure injury healing within home care settings, with the goal of identifying the most efficient and cost-effective treatment protocol in a long-term follow-up.

## 2 Methods

### 2.1 Ethical considerations

This retrospective case study was conducted in accordance with the ethical standards of the institutional and/or national research committee and with the 1964 Helsinki Declaration and its later amendments or comparable ethical standards. Ethical approval was obtained from the Institutional Review Board (IRB, 1130503) of Chimei Medical Center, which waived the requirement for informed consent owing to the non-interventional design of the study and the use of de-identified patient data. All patient data were extracted from electronic health records and anonymized prior to analysis to ensure confidentiality and compliance with data protection regulations. The research team had access to only de-identified data and maintained the privacy and security of patient information throughout the study.

### 2.2 Study design

In this retrospective observational study, we reviewed the medical records of patients with pressure ulcers who received l-arginine supplementation between June and December 2023. Based on the documented treatment plans, patients were classified into two cohorts according to their l-arginine dosage: 7 g per day (low-dose) or 14 g per day (high-dose). Initial dosage assignments were determined through a structured clinical assessment process involving both the attending physician and registered dietitian, who considered multiple factors, including nutritional status, wound severity, and overall health condition. The healthcare team implemented these protocols following the institution’s established clinical guidelines for home-care wound management.

### 2.3 Inclusion and exclusion criteria

Study participation was limited to physically disabled patients aged 18 years or older who presented with grade 3 or 4 pressure injuries according to the National Pressure Ulcer Advisory Panel (NPUAP) staging system (19). We excluded patients whose pressure wounds had persisted for >12 months, those with frequent hospital readmissions due to wound infections, and individuals diagnosed with osteomyelitis or diabetic foot ulcers, as these conditions could complicate the assessment of healing outcomes. Additionally, patients prescribed medications known to interfere with wound healing were excluded from the analysis. This included individuals receiving hydroxyurea or those with documented daily use of systemic corticosteroids exceeding 10 mg of prednisolone or 1.5 mg of dexamethasone (20).

### 2.4 Care for pressure ulcers

At our institute, patients received Abound™ (Abbott, United States), a specialized nutritional supplement containing l-glutamine, l-arginine, citric acid, and calcium HMB. Each package contained 7 g of l-arginine, and patients were prescribed either one package (7 g) or two packages (14 g) daily based on clinical judgment by attending physicians and dietitians. All nursing care was provided uniformly, independent of the patient’s nutritional supplement intake. All patients received standardized wound care according to our institutional home care protocol. Modern moist wound healing dressings, including hydrocolloid, foam, and alginate, were selected based on the exudate level of the wound. Silver-containing dressings were used in cases of suspected local infection. Conventional gauze dressings were used only when specifically indicated, such as in wounds with minimal exudate or when preferred by the patient. Nursing care included regular wound cleansing, appropriate dressing selection based on wound characteristics, and dressing changes at predetermined intervals. Care protocols incorporated evidence-based practices, including proper positioning techniques, use of pressure-relieving devices, and topical treatments as needed. Patient and caregiver education focuses on the fundamentals of wound care and early detection of complications.

### 2.5 Outcome measurement and data collection

Pressure ulcers were assessed using DESIGN-R wound assessment scores, a validated tool (content validity index: 0.93; Cronbach’s alpha: 0.70–0.79) that evaluates wound depth, exudate, size, inflammation/infection, granulation tissue, necrotic tissue, and pocket formation. The composite DESIGN-R score ranged from 0 to 71 points, with higher scores indicating greater wound severity (21). These assessments were conducted during routine care at baseline and approximately every 2 weeks thereafter. A trained research nurse extracted data, including pressure injury onset dates and healing progression, from electronic health records.

### 2.6 Statistical analysis

Based on the anticipated moderate effect size (Cohen’s d between 0.5 and 0.8), we calculated an initial sample size requirement of 100 subjects, using a power of 0.80, and an alpha level of 0.05. To account for the potential data loss and variability inherent in retrospective analyses, we increased the sample size by 20%, resulting in a final target of 120 participants. Baseline demographic characteristics and wound parameters were compared between the groups using chi-square tests for categorical variables and independent t-tests for continuous variables. Statistical analyses were performed using SPSS version 26.0 to assess the effectiveness of l-arginine supplementation on pressure ulcer healing. Generalized Estimating Equations (GEE) were used as the primary analytical approach, selected for their ability to accommodate longitudinal data, account for correlations within repeated measures, and adjust for potential confounders and missing values (22–24). A GEE model with an identity link function and exchangeable correlation structure was applied to evaluate changes in DESIGN-R scores over time. Treatment group, time, and their interaction were included as fixed effects, while covariates such as age, sex, and baseline wound severity were adjusted for. Healing trajectories between the low- and high-dose groups were compared across multiple time points, including baseline, 1, 4, and 6 months.

## 3 Results

### 3.1 Patient selection and baseline characteristics

**Figure 1:**
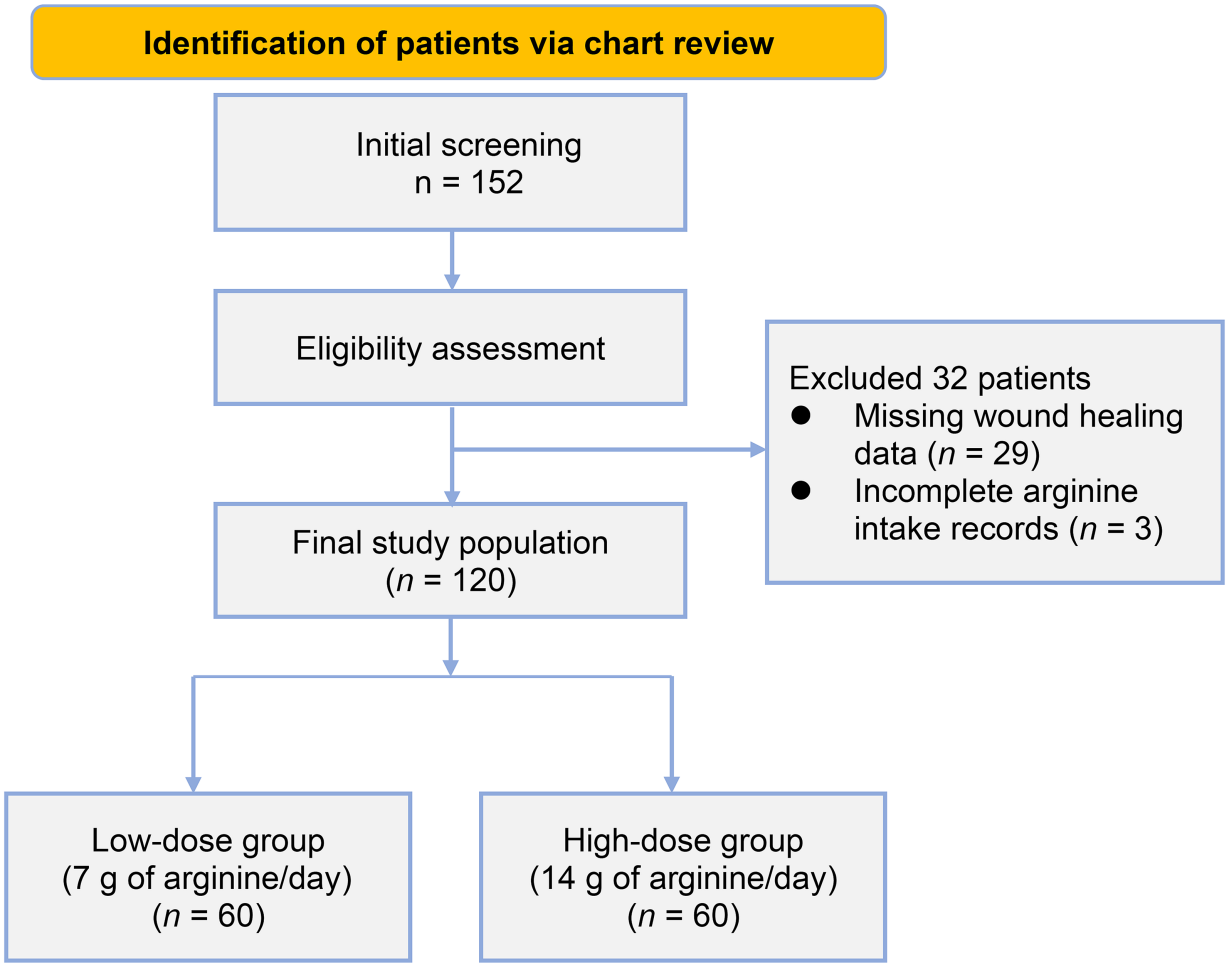
Flowchart of patient selection.

A total of 120 patients with grade 3 or 4 pressure injuries were included in the analysis, with 60 patients in each treatment group (Figure 1). The baseline characteristics of both groups are presented in Table 1. The majority of patients in both groups were old adults (≥65 years), comprising 75% of the low-dose group and 90% of the high-dose group (*p* = 0.82). Both groups exhibited comparable body mass index (BMI), with means of 14.56 ± 1.79 kg/m^2^ in the low-dose group and 14.62 ± 1.82 kg/m^2^ in the high-dose group (*p* = 0.67), indicating significant malnutrition across the study population.

**Table 1:** Baseline characteristics of patients.

Variables	Low-dose group	High-dose group	*p*-value
	(*N* = 60)	(*N* = 60)	
Age (years)			0.82
≥65	45 (75%)	54 (90%)	
<64	15 (25%)	6 (10%)	
BMI (kg/m^2^)	14.56 (1.79)	14.62 (1.82)	0.67
Sex			0.35
Male	26 (43%)	20 (33%)	
Female	34 (57%)	40 (67%)	
Albumin (g/dL)	2.31 (0.47)	2.33 (0.47)	0.58
Healing Time for Pressure Injuries (day)	72.76 (59.27)	82.23 (72.66)	0.46
Number of comorbidities^†^			0.06
≤2	22 (37%)	12 (20%)	
≥3	38 (63%)	48 (80%)	
Degree of disability^⁋^			0.16
Total dependence	6 (10%)	18 (30%)	
Partial dependence	54 (90%)	42 (70%)	
Caregiver			0.41
Family	50 (83%)	50 (83%)	
Foreign domestic helper	10 (17%)	10 (17%)	

^†^Comorbidities assessed included hypertension, diabetes mellitus, ischemic heart disease, heart failure, chronic kidney disease (CKD), dementia, and Parkinson’s disease; low-dose group: 7 g of arginine/day; high-dose group: 14 g of arginine/day. ^⁋^Total dependence was defined as complete immobility requiring full caregiver assistance for all activities of daily living, whereas partial dependence was defined as the ability to perform some activities of daily livings, independently with functional limitations.

**Figure 2:**
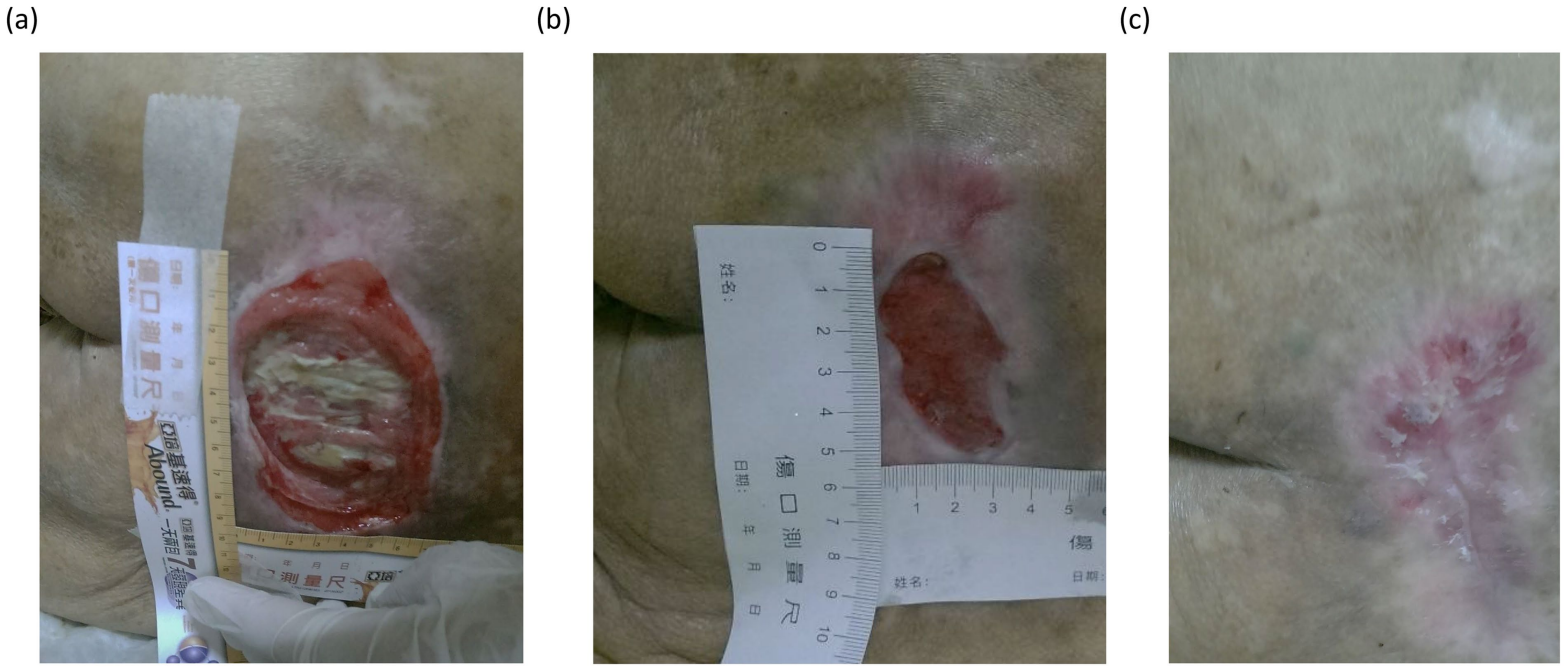
Sequential images underscored the typical healing trajectory observed in patients receiving l-arginine supplementation. Panel **(a)** shows the initial appearance of severe pressure injury, characterized by extensive tissue necrosis and slough formation. In the intermediate phase **(b)**, substantial granulation tissue development and wound contraction were observed, accompanied by a reduction in wound size and surrounding inflammation. In the final stage **(c)**, the wound demonstrated significant epithelialization and remodeling, with only minor residual scarring.

The sex distribution showed a slight female predominance in both groups (57% vs. 67%, *p* = 0.35). Serum albumin levels were similarly low in both groups (2.31 ± 0.47 g/dL vs. 2.33 ± 0.47 g/dL, *p* = 0.58), further reflecting the compromised nutritional status of the study population. The average healing time for pressure injuries was 72.76 ± 59.27 days in the low-dose group compared to 82.23 ± 72.66 days in the high-dose group, though this difference did not reach statistical significance (*p* = 0.46). Representative wound healing progressions are illustrated in Figure 2. Regarding the degree of disability, the majority of patients in both groups had partial dependence (90% vs. 70%, *p* = 0.16), with the remainder classified as totally dependent. Among those with total dependence, 66.7% in the low-dose group and 38.9% in the high-dose group had documented neurological impairments, including post-stroke sequelae, advanced dementia, and Parkinson’s disease, which contributed to their immobility. Caregiving was predominantly provided by family members (83% in both groups), with foreign domestic helpers caring for the remaining 17% of the patients in each group (*p* = 0.41).

### 3.2 Pressure injury site distribution

The anatomical distribution of pressure injuries varied significantly between the two groups, as shown in Table 2. The sacrum (midline) was the most common site in both groups, affecting 55.0% of the patients in the low-dose group and 51.7% in the high-dose group (*p* = 0.714). However, significant between-group differences were observed in several other anatomical locations. The right hip was more frequently affected in the low-dose group (26.7% vs. 8.3%, *p* = 0.008), as was the left back (20.0% vs. 3.3%, *p* = 0.005). Conversely, the left heel was more commonly affected in the high-dose group (30.0% versus 11.7%, *p* = 0.013), as were the unspecified sacral regions (21.7% versus 8.3%, *p* = 0.041).

**Table 2:** Distribution of pressure injury sites at baseline between low-dose and high-dose l-arginine supplementation groups.

Pus part	Low-dose group	High-dose group	*p*-value
	(*n* = 60)	(*n* = 60)	
Sacrum, midline	33 (55.0%)	31 (51.7%)	0.714
Hip, right	16 (26.7%)	5 (8.3%)	0.008
Hip, left	15 (25.0%)	20 (33.3%)	0.315
Back, left	12 (20.0%)	2 (3.3%)	0.005
Sacrum, unspecified	10 (16.7%)	17 (28.3%)	0.126
Buttock, right	9 (15.0%)	12 (20.0%)	0.471
Heel, left	7 (11.7%)	18 (30.0%)	0.013
Buttock, left	6 (10.0%)	7 (11.7%)	0.769
Buttock, midline	6 (10.0%)	5 (8.3%)	0.752
Sacrum, other unspecified	5 (8.3%)	13 (21.7%)	0.041

### 3.3 Pressure ulcer assessment and healing

The progression of wound healing, as measured by DESIGN-R scores, was analyzed using Generalized Estimating Equations (GEE) and is presented in Table 3. Overall, both groups demonstrated significant improvement in DESIGN-R scores over the six-month follow-up period, indicating progressive wound healing regardless of l-arginine dosage.

**Table 3:** GEE analysis of wound healing measured by DESIGN-R score in patients receiving arginine 7 g/d or 14 g/d supplementation.

Parameter	Estimate (B)	Standard error (SE)	Significance (p)
Basic parameters			
Intercept	26.39	0.78	<0.001
Group (arginine 7 g vs. 14 g)	0.37	1.11	0.737
Time effect (Change in DESIGN-R Score)			
1 month after receiving the case	−6.35	0.39	< 0.001
2 months after receiving the case	−7.12	0.59	< 0.001
3 months after receiving the case	−9.42	0.79	< 0.001
4 months after receiving the case	−10.76	0.95	< 0.001
5 months after receiving the case	−16.4	1.28	< 0.001
6 months after receipt of the case	−23.75	1.19	< 0.001
Interaction effects (additional effect of 7 g group relative to 14 g group)			
Group (arginine 7 g) × time (1 month)	−6.42	0.83	< 0.001
Group (arginine 7 g) × time (2 months)	−2.42	1.15	0.036
Group (arginine 7 g) × time (3 months)	−1.34	1.39	0.334
Group (arginine 7 g) × time (4 months)	−1.35	1.46	0.353
Group (arginine 7 g) × time (5 months)	−0.15	1.66	0.929
Group (arginine 7 g) × time (6 months)	−3.85	1.52	0.011

Negative values indicate decrease in DESIGN-R scores, representing improvement in wound healing; lower scores indicate better healing outcomes. Statistically significant values (*p* < 0.05) indicate meaningful differences between groups.

The baseline DESIGN-R scores were comparable between the groups (intercept estimate = 26.39, SE = 0.78, *p* < 0.001), with no significant difference between the low-dose and high-dose groups (estimate = 0.37, SE = 1.11, *p* = 0.737). Both groups showed significant improvements in DESIGN-R scores at each time point compared with baseline, with the magnitude of improvement increasing over time.

Interaction effect analysis revealed significant between-group differences in healing trajectories. The low-dose (7 g) group showed significantly better improvement at 1 month compared to the high-dose group (*B* = −6.42, SE = 0.83, *p* < 0.001), indicating a more rapid initial healing response with the lower dose. This advantage continued for 2 months, although with a smaller magnitude (*B* = −2.42, SE = 1.15, *p* = 0.036). During the middle period of the study (3–5 months), the between-group differences were not statistically significant (*p* > 0.05). However, by 6 months, the low-dose group again demonstrated significantly better outcomes than the high-dose group (*B* = −3.85, SE = 1.52, *p* = 0.011).

These results suggest a time-dependent effect of l-arginine dosage on pressure injury healing, with the 7 g daily dose demonstrating superior efficacy during both the early (1–2 months) and late (6 months) phases of the healing process. The observed temporal pattern of the healing response indicates that the lower dose of l-arginine may offer advantages in both initiating the healing process and promoting long-term wound resolution compared to the higher dose regimen.

## 4 Discussion

The findings of this study provide valuable insights into the role of l-arginine supplementation in the management of pressure ulcers in home care settings. Our results reveal that while both 7 g and 14 g doses of l-arginine promote wound healing, the 7 g dose demonstrated superior efficacy in the early stages of treatment. This was evidenced by a significant reduction in DESIGN-R scores at 1 month post-treatment initiation, with a decrease of 6.42 points (*p* < 0.001) compared to the 14 g dose. The healing benefits of the 7 g dose were also particularly notable at the six-month mark, where a significant improvement of 3.85 points (*p* = 0.011) was observed. These results align with previous research highlighting the crucial role of l-arginine in tissue repair, immune function enhancement, and collagen synthesis, which are all essential components of the wound healing process.

Our GEE analysis revealed a complex healing response, where lower doses offer substantial early- and late-stage benefits, but the healing trajectory may plateau or fluctuate over time. The lack of significant differences between the 7 g and 14 g doses across most time points indicates that higher doses may not provide additional benefits in wound healing. This observation is particularly relevant for clinical practice in geriatric care, where optimizing nutritional supplementation without excessive dosing becomes increasingly important due to age-related changes in metabolism and the potential for adverse effects. The non-linear healing pattern observed in the 7 g group, with significant improvements at 1 and 6 months but diminished effects at 4 months, may be explained by several physiological mechanisms. This temporal effect could reflect the dose-dependent actions of l-arginine at different wound healing phases. Lower doses may provide optimal substrates for NO production during the early inflammatory phase and later remodeling phase, while avoiding potential competitive inhibition of other amino acid transport or enzyme saturation that might occur with higher doses (15, 25). Additionally, the wound microenvironment undergoes substantial changes throughout the healing process, potentially altering the efficacy of l-arginine supplementation at different time points (26). A previous study suggested that prolonged high-dose amino acid supplementation may trigger adaptive metabolic responses that temporarily reduce effectiveness until homeostatic adjustments occur [38]. However, these explanations remain speculative, as the precise mechanisms underlying the temporal efficacy pattern of different l-arginine doses in pressure injury healing require further investigation through controlled prospective studies.

Our findings align with earlier studies indicating that lower doses of l-arginine (4.5 g-9 g) may be sufficient for improving wound healing outcomes (27, 28). A previous study (17) demonstrated similar improvements with a 4.5 g daily dose compared to 9 g, suggesting that moderate supplementation provides adequate substrate for nitric oxide production and collagen synthesis. While a recent meta-analysis (18) suggested enhanced pressure injury healing with arginine supplementation exceeding 15 g daily, their conclusion was based on only two small-scale studies with brief follow-up periods. Our extended six-month follow-up provide stronger evidence supporting the efficacy of lower doses. The documented role of l-arginine as a precursor to nitric oxide, promoting vasodilation and enhancing blood flow to wound sites, appears to be adequately fulfilled with the 7 g dose, without requiring the higher 14 g dose that could potentially increase the risk of adverse effects, particularly in patients with compromised renal function.

This study underscores the importance of personalized nutritional interventions, particularly in home care settings where resources are limited and patient needs are highly individualized. The observed variability in the healing response suggests that a “one-size-fits-all” approach to l-arginine supplementation may not be appropriate. Instead, clinicians should consider a tailored strategy that considers patient-specific factors such as baseline nutritional status, comorbid conditions, and wound severity. Our findings suggest that a 7 g daily dose of l-arginine represents an optimal starting point for most patients. Nevertheless, we propose dose adjustment based on individual response as a potential strategy for future clinical practice. The sustained effectiveness of supplementation over 6 months highlights the importance of continuing treatment throughout the entire healing process, rather than discontinuing after initial improvements are observed. This long-term approach is particularly relevant in home care settings, where pressure injuries often require extended treatment periods.

From an economic perspective, the potential for lower doses of l-arginine to yield similar or better healing outcomes than higher doses represents a significant opportunity for cost savings in home care settings. By accelerating the healing process, particularly in the early stages of treatment, low-dose supplementation can reduce the need for more frequent dressing changes and the risk of complications such as infections (16, 29). These benefits translate to decreased healthcare utilization and reduced financial burden on both healthcare systems and patients. In resource-limited home care environments, such cost-effectiveness considerations are paramount for ensuring sustainable care delivery while optimizing patient outcomes.

This study has several limitations that warrant consideration. First, although we applied statistical adjustments to account for baseline differences, treatment allocation was determined by clinical judgment rather than randomization, introducing potential selection bias. As a result, unmeasured confounding variables may have influenced the observed outcomes. Future prospective randomized controlled trials are warranted to confirm the causal relationship between l-arginine dosage and pressure injury healing. Second, another potential limitation is the significant difference in anatomical site distribution between the two groups at baseline. Wound location can influence healing through factors such as localized pressure, tissue perfusion, and ease of pressure relief. These site differences may have contributed to the observed outcomes independently of the l-arginine dosage. Future studies should consider controlling for wound location through stratified randomization or site-specific subgroup analyses. Third, it should be noted that the nutritional supplement used in this study (Abound™) contained not only l-arginine, but also l-glutamine and β-hydroxy-β-methylbutyrate (HMB), both of which have documented benefits for wound healing. Therefore, the improvements observed were likely due to the combined effects of these ingredients, and the independent contribution of l-arginine could not be determined from our data. Future studies employing single-nutrient formulations or factorial designs are warranted to clarify the specific role of l-arginine in pressure injury management. Fourth, additional studies should explore the potential synergistic effects of combining l-arginine with other nutrients, such as zinc, vitamin C, and omega-3 fatty acids, which have demonstrated complementary roles in wound healing. A more detailed investigation of the temporal effects of l-arginine supplementation could help optimize dosing schedules and explain the observed fluctuations in healing response over time. Finally, although compliance was indirectly monitored through home care nursing records and caregiver reports, these data were not systematically collected or quantified. Given that the study population was severely malnourished, variations in dietary intake and supplementation adherence may have influenced healing outcomes.

## 5 Conclusion

This study supports the efficacy of l-arginine supplementation in promoting wound healing in home care patients with pressure ulcers, with particular emphasis on the benefits of a 7 g daily dose. The time-dependent effects observed highlight both early and late-stage healing advantages of the lower dose, without evidence of additional benefits from the higher 14 g dose. These findings have significant implications for clinical practice, suggesting that low-dose l-arginine supplementation offers a cost-effective approach to pressure ulcer management in home care settings. The consistent healing benefits observed over the six-month study period reinforce the importance of continued nutritional support throughout the healing process. As healthcare systems worldwide face resource constraints, especially in home care environments, optimizing treatment regimens to maximize both clinical and economic outcomes has become increasingly crucial. Future research should build on these findings to further refine personalized nutritional interventions for pressure ulcer management, ultimately improving the quality of life for affected patients while reducing the associated healthcare burden.

## Data Availability

The raw data supporting the conclusions of this article will be made available by the authors, without undue reservation.
